# Supplementary material for: Effect of Tecoma stans (L.) Juss. ex Kunth in a Murine Model of Metabolic Syndrome
Source: Plants (Basel). 2022 Jul 7;11(14):1794. doi: 10.3390/plants11141794 (PMC9324241; doi:10.3390/plants11141794)
Supplement: Supplementary file 1 [file plants-11-01794-s001.zip › plants-1779869-supplementary.pdf]

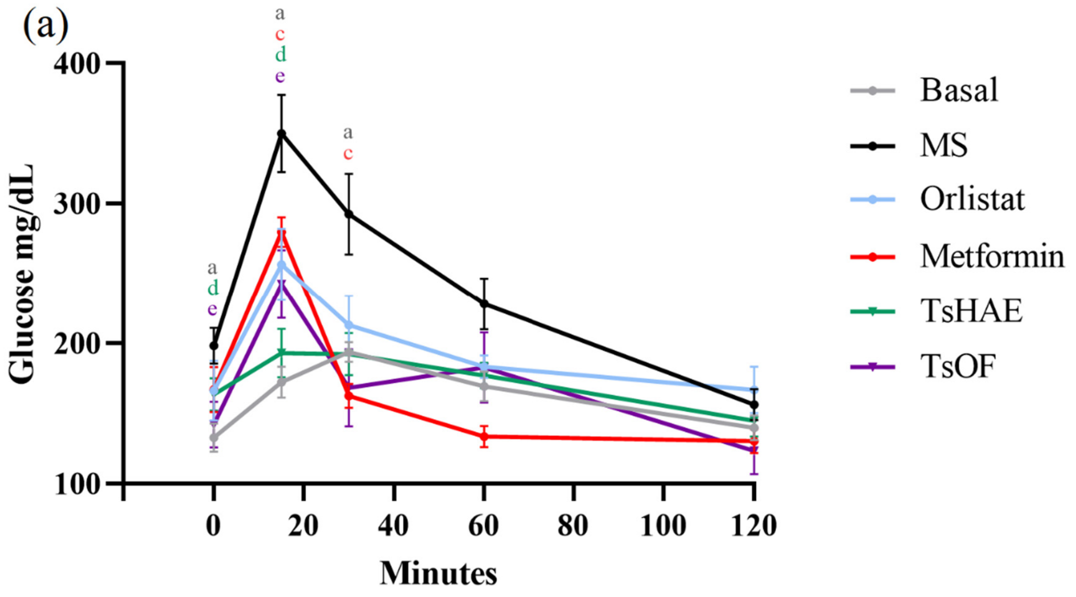


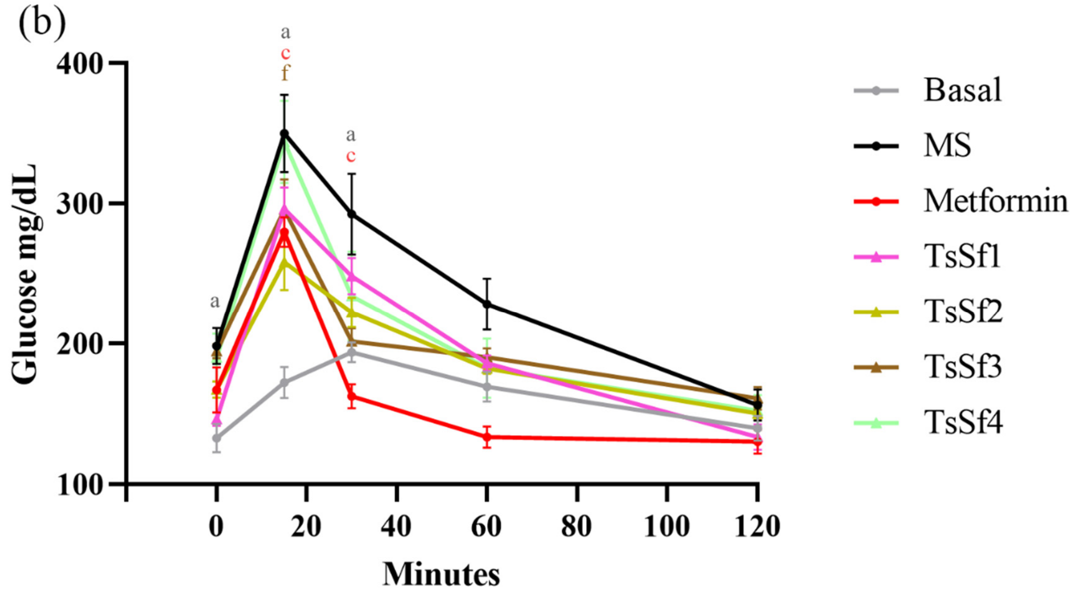


**Figure S1.** Glucose tolerance test in mice fed with standard diet or hypercaloric diet after ten weeks of treatment. (a) Shows hydroalcoholic extract and organic fraction of *T.stans* compared to control groups. (b) Shows subfractions obtained from the organic fraction of *T. stans* (TsSf1, TsSf2, TsSf3 and TsSf4) compared to control groups. Data are expressed as mean ± SEM, n=6 in each group. One-way ANOVA-RM, followed by Tukey's test. $^{a}$ $p < 0.05$ by comparison of MS with Basal. $^{c}$ $p < 0.05$ by comparison of Metformin with Basal. $^{d}$ $p < 0.05$ by comparison of TsHAE with Basal. $^{e}$ $p < 0.05$ by comparison of TsOF with Basal. $^{f}$ $p < 0.05$ by comparison of TsSF1 with Basal.
